# IL-1β Promotes *Staphylococcus aureus* Biofilms on Implants *in vivo*

**DOI:** 10.3389/fimmu.2019.01082

**Published:** 2019-05-17

**Authors:** Rodrigo Gutierrez Jauregui, Henrike Fleige, Anja Bubke, Manfred Rohde, Siegfried Weiss, Reinhold Förster

**Affiliations:** ^1^Institute of Immunology, Hannover Medical School, Hannover, Germany; ^2^Central Facility for Microscopy, Helmholz Center for Infection Research, Braunschweig, Germany; ^3^RESIST, Cluster of Excellence 2155, Hannover Medical School, Hannover, Germany

**Keywords:** biofilm, *Staphylococcus aureus*, IL1β, NETs, osmotic pump

## Abstract

Implant associated infections represent a serious health burden in clinics since some microorganisms are able to colonize biological surfaces or surfaces of indwelling medical devices and form biofilms. Biofilms represent communities of microorganisms attached to hydrated surfaces and enclosed in self-produced extracellular matrix. This renders them resistant to exogenous assaults like antibiotics or immune effector mechanisms. Little is known regarding the role of the immune system in the formation of biofilms during implant associated infections, largely due to the lack of suitable mouse models. Here we use colonized osmotic pumps in mice to study the interaction of an activated immune system with biofilm-forming *Staphylococcus aureus* encoding Gaussia luciferase. This approach permits biofilm formation on the osmotic pumps in living animals. It also allows the continuous supply of soluble immune cell activating agents, such as cytokines to study their effect on biofilm formation *in vivo*. Using non-invasive imaging of the bioluminescent signal emitted by the lux expressing bacteria for quantification of bacterial load in conjunction with light and electron microscopy, we observed that pump-supplied pro-inflammatory cytokine IL-1β strongly increased biofilm formation along with a massive influx of neutrophils adjacent to the biofilm-coated pumps. Thus, our data demonstrate that immune defense mechanisms can augment biofilm formation.

## Introduction

More than 4.2 million healthcare-associated infections occur every year in European long-term care facilities, of which 40% to 70% are due to indwelling medical devices (1, 2). Depending on the device, the mortality rate can vary from below 5% for devices, such as dental implants, to more than 25% for mechanical heart valves. Since antibiotics treatment alone is ineffective to treat these infections, the only possible treatment is the removal of the infected device followed by application of antibiotics (3).

The inefficacy of antibiotic treatment is due to the formation of biofilms by microorganisms colonizing the surface of the implants. Biofilms are arrangements of microorganisms enclosed in extracellular matrices and attached to hydrated surfaces. Extensive studies have been undertaken to better understand and interfere with the ability of Gram-positive and -negative bacteria to form biofilms (4, 5) These studies identified the effect of quorum sensing i.e., the presence of molecules, receptors as well as signaling cascades (6, 7) that allow the bacteria to monitor their aggregation status and spark biofilm formation *in vivo* (8–13). In addition, biofilm extracellular matrices have been investigated. These are mainly composed of polysaccharides but also might contain peptides and/or DNA which can determine a biofilms virulence or toxicity (14–16). Biofilms present considerable therapeutic challenges, since these bacteria exhibit altered growth rates, gene expression profiles, and protein synthesis. Embedded in the extracellular matrix, bacteria become resilient to both antibiotics and effector mechanisms of the host's immune system (17–20). These infections are virtually untreatable and completely resistant to present therapies, despite intensive studies to identify some of the mechanisms underlying the inefficacy of antibiotic treatment. For instance, in *Salmonella enterica* (21, 22) and *Escherichia coli* (23, 24) biofilm curli amyloids have been identified as the extracellular matrix component that promotes bacterial resistance against antibiotics and immune effectors. To counteract this resistance, potential treatment schedules of *Pseudomonas aeruginosa* biofilm infections were developed by altering antibiotic administration regimes after real time imaging analysis revealed antibiotic dependent bacterial killing and regrowth kinetics (25).

The formation of biofilms has been considered a defense mechanism against environmental and immune effector mechanisms. For instance, it has been shown that *Salmonella enterica serovar Typhimurium* produce biofilms when prompted by nutrient starvation or oxygen tension (26). Similarly, in a mouse model, *S. Typhimurium* forms biofilms when colonizing tumors. These structures were absent in animals when neutrophils were depleted (27, 28).

*Staphylococcus aureus* is a highly relevant bacterium associated with implant infections. It is a Gram-positive bacterium responsible for the majority of skin and soft tissue infections in humans. Although *S. aureus* infections usually originate in the skin, invasive and frequently life-threatening infections are common consequences in implant associated infections. In addition, many community and hospital acquired *S. aureus* infections are complicated by virulent methicillin and multidrug-resistant strains. Although *S. aureus* are often found to colonize medical devices and form biofilms *in vivo* little is known on the role of the immune system in the formation of *S. aureus* implant-associated biofilms (29–31). However, for the rational design of treatment strategies, this would be an essential asset.

Several groups have tested the reactions of the immune system to bacterial biofilms using mouse models of subcutaneously implanted catheters. In the case of *S. epidermidis* biofilms increased expression of the immune activating cytokines TNFα, IL-6, IL-10, IL-1β, and IFNγ in tissues surrounding the colonized inserts have been reported (32, 33). Surprisingly, IL-1β could be shown to support survival of the bacteria under these conditions.

In contrast, *S. aureus* biofilms located inside catheters often result in downregulation of IL-1β by macrophages (34). Functional changes in macrophages and neutrophils have also been reported in this model including shifts from pro-inflammatory to anti-inflammatory macrophages located near *S. aureus* biofilms and recruitment of neutrophils which exhibit severely reduced chemotaxis and increased production of oxygen radicals (35, 36). This phenotype was associated with persistence of *S. aureus* biofilms.

Based on these findings, we put forward the hypothesis that the *in vivo* development of biofilms by these bacteria depend on intrinsic propensities of the immune system. To test this hypothesis, we developed a novel mouse model to study the interaction of an activated immune system with biofilm-forming *S. aureus*. This model is based on implanting C57Bl/6 mice subcutaneously with osmotic pumps colonized by *Staphylococcus aureus* Xen29. The osmotic pumps allow the continuous release of immune modulating substances that help to address their effect on the formation of biofilms. Using this model we observed that the pro-inflammatory cytokine IL-1β, promotes early spread of *S. aureus* on the surface of the implants as well as increased colonization of other organs. Furthermore, we observed that recruitment of neutrophils to the implant area provided protection against early bacterial colonization of peri-implant tissues.

## Materials and Methods

### Strains and Growth Media

*Staphylococcus aureus* Xen29 (Perkin Elmer, GenBank accession L36472.1) bacteria were streaked onto Blood agar (BA) plates [premixed blood agar medium powder (Oxoid), water, sheep's blood (Oxoid)] and grown overnight (16 h) at 37°C in a bacterial incubator. Single colonies were picked and cultured in Trypticase soy broth (TSB) at 37°C in a shaking incubator (240 rpm) overnight, followed by a 1:20 subculture at 37°C overnight to obtain midlogarithmic phase bacteria. OD600 was measured to estimate the number of colony forming units (CFU), which was verified after overnight culture on BA plates.

### Animal Model

Eight- to twelve-week-old female C57BL/6 mice were randomly assigned to experimental groups for use in all experiments. The mice were obtained from Envigo (Germany) and were single housed and maintained under pathogen-free conditions. All experiments were conducted in accordance with the local animal welfare regulations reviewed by the institutional review board and the Niedersächsisches Landesamt für Verbraucherschutz und Lebensmittelsicherheit (LAVES) under the permission number 14–1567.

### *In vitro* Bacterial Device Colonization

Osmotic pumps (ALZET 1007D) were sterilized with 70% ethanol and air dried before colonization. Bacterial colonization was established on the pumps by incubating them in tubes containing 5.0 ml of a *S. aureus* cell suspension (10^8^ CFU/ml) in TSB in the phase of exponential growth. After incubation for 24 h at 37°C, colonized pumps were recovered with sterile forceps and rinsed once with TSB to remove unbound bacteria. Pumps where then filled with 100 μl of one of the following substances: IL-1β (83 μg/ml) (37), IL-6 (83 μg/ml) (38, 39), IL-10 (166 μg/ml) (40), IL-12 (83 μg/ml) (41), IL-17 (125 μg/ml) (42), IL-23 (166 μg/ml) (43), IFNγ (83 μg/ml) (44), TNFα (166 μg/ml) (45), anti-TGF-β1 (166 μg/ml) (46, 47), anti-IL-1β (150 μg/ml) (48), or PBS. All the concentrations of cytokines and antibodies used were based on values found in the literature.

### Implantation of Colonized Pumps

Mice were anesthetized by injection of Ketamin (50 mg/kg body weight) and Xylazin (10 mg/kg body weight), before a skin incision was made between the shoulder blades, and an osmotic pump was implanted in the subcutaneous space. The wound was closed with surgical staples. Mice (*n* = 6 mice per experimental condition) were monitored at least every other day for the duration of the experiments.

### Neutrophil Depletion

Mice were given intraperitoneal injections of 100 μg of anti-Gr-1 mAb (clone RB6-8C5) 1 day before pump implantation and on every third day thereafter. PBS injections were administered to control mice.

### *In vivo* Bioluminescent Imaging

*In vivo* bioluminescent imaging was performed on anesthetized mice (2% isoflurane) using a Spectrum CT IVIS *in vivo* imaging system. Briefly, photons emitted from *S. aureus Xen29* were collected during a 30-s exposure using the IVIS Imaging System. Bioluminescent images were displayed using a pseudocolor scale (blue representing the least-intense light and red representing the most-intense light) that was overlaid on a gray-scale image to generate a two-dimensional picture of the distribution of bioluminescent bacteria in the animal. The acquired image data were saved as two-dimensional arrays containing values corresponding to the number of photons contained within each pixel as measured within a region of interest using Living Image software. The bioluminescent signals detected from the pump implanted site were measured and correlate to the actual bacterial load measured by the *ex vivo* CFU on the pumps.

### *Ex vivo* CFU Counting

Mice were killed on day 12 post-infection, and the heart, liver, spleen and draining lymph nodes, as well as the osmotic pumps, were harvested. Bacterial CFU were isolated by homogenizing the tissue in PBS on ice or in the case of the osmotic pumps, sonicated (JP-890, Skymen) for 30 min in PBS at a frequency of 40 kHz. We did not observe any influence of this procedure in the viability of the bacterial cells. *Ex vivo* CFU were counted after serially diluted tissue homogenates and suspended PBS were plated overnight on BA plates.

### H&E Staining

Formalin fixed, paraffin embedded tissue blocks were prepared from mouse skin 2 days post-infection as follows: Tissue samples were fixed in 4% paraformaldehyde overnight, dehydrated using increasing concentrations of acetone, embedded in paraffin, and prepared for histological analysis. For H&E staining, 2-micron sections were stained using routine methods. Briefly, the paraffin was removed from sections with xylenes, after which they were washed in ethanol and then rehydrated in distilled water. Sections were incubated 10 min at room temperature in Hematoxylin and washed thrice with water. Excess stain was removed by washing with 30% ethanol + 1% HCl and rinsing with water. After two steps of ethanol washing the cytoplasm was stained by incubation in 70% ethanol and 1% Eosin for 10 s. Finally, sections were washed in ethanol and xylenes.

### Immunohistochemical Analysis

Formalin fixed, paraffin embedded tissue blocks were prepared from mouse skin 2 days post-infection as follows: Tissue samples were fixed in 4% paraformaldehyde overnight, embedded in paraffin, and prepared for histological analysis. For immunohistochemistry, 2-micron sections were immune-labeled using routine immunoperoxidase methods. Briefly, the paraffin was removed from sections with xylenes, after which they were washed in ethanol and then rehydrated in PBS. Samples were blocked 30 min in 3% H_2_O_2_ solution (Sigma-Aldrich), blocked with 10% goat serum/TRIS-buffered saline for 15 min, incubated for 2 h at room temperature with the primary antibodies, incubated 1 h with species-specific secondary antibodies and lastly developed in 3,3′-diaminobenzidine tetra hydrochloride as a chromogen. Primary antibodies used: anti-Myeloperoxidase (rabbit 1:50 ab9535, Abcam) and anti-histone H3 (citrulline R2 + R8 + R17) (rabbit 1:50 ab5103). Secondary antibody used: Goat-anti-rabbit IgG (H&L) mouse/human ads-HRP (1:200, Southern Biotech).

### Light-Sheet Microscopy

Explanted osmotic pumps were fixed with 4% PFA and stained for 1 h on ice with F(ab′)_2_-Cy5 from anti-*S. aureus* antibody (1:500, Abcam ab37644). Samples were then washed in PBS. Osmotic pumps were imaged in water, using an UltraMicroscope II (LaVision BioTec). During imaging, specimens were excited with a 488-nm light for detection of autofluorescence and a 647-nm light for the detection of the Cy5-conjugated antibody. A step size of 10 μm was used to collect images. Image analysis was performed using Imaris software (Bitplane). TIFF files collected from the light sheet microscope were reconstructed into representative 3D images.

### Field Emission Scanning Electron Microscopy

Samples were fixed with 2% glutaraldehyde and 5% formaldehyde in HEPES buffer (HEPES 0.1 M, 0.09 M sucrose, 10 mM CaCl_2_, 10 mM MgCl_2_, pH 6.9) and kept at 7°C. After washing with TE-buffer (20 mM TRIS, 2 mM EDTA, pH 7,0) for 10 min at room temperature samples were dehydrated in a graded series of acetone (10, 30, 50, 70, 90%) on ice for 30 min for each step. After adjustment to room temperature samples were further dehydrated with 100% acetone before subjected to critical-point drying with liquid CO_2_ (CPD030 Bal-Tec, Liechtenstein). Dried samples were covered with a gold-palladium film by sputter coating (SCD 500 Bal-Tec, Liechtenstein) before examination in a Zeiss field emission scanning electron microscope (FESEM) Merlin (Oberkochen, Germany) using the Everhart Thornley SE-detector and the inlens SE-detector in a 75:25 ratio with an acceleration voltage of 10 kV. Contrast and brightness were adjusted with Adobe Photoshop CS5.

### Quantification and Statistical Analysis

GraphPad PRISM 8 was used to determine average values and standard deviations. All the error bars represent the SD. For each figure, the number of replicates is *n* = 6, all other information relevant for assessing the accuracy and precision of the measurements is included in the corresponding legend. Multiple *t*-tests were performed with GraphPad PRISM 8 using the Holm-Sidak method (alpha = 0.05), for comparisons between data for *S. aureus* colonized pumps containing PBS and all other cytokines (^*^p < 0.0332, ^**^p < 0.0021, ^***^p < 0.0002).

## Results

### A Novel Mouse Model to Study *Staphylococcus aureus* Biofilms *in vivo*

**Figure 1:**
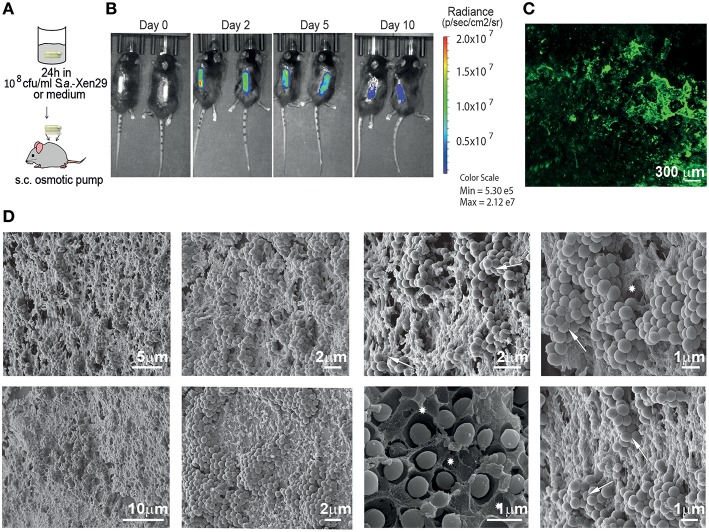
Mouse model for implant-associated infection. **(A)** Experimental setup. **(B)** Biofilm implant-associated infection caused by *S. aureus* Xen29 in C57Bl/6 mice was monitored in real time for 10 days. Images were taken every 24–48 h. Mice were anesthetized with isoflurane during imaging procedures. Luminescent counts were collected for an exposure time of 30 s within each measured region of interest (ROI) and quantified with Living Image software. **(C)** Representative Light Sheet micrograph from the surface of immunostained osmotic pumps post-explantation, showing anti-*S aureus* antibody in green. **(D)** Images recorded by scanning electron microscopy of colonized implant surfaces. Excavations with the shape of bacterial cells could be found in the matrix (asterisks) as well as bacterial cells with altered geometry (arrows). These are representative images for six mice under the same experimental conditions.

To evaluate the suitability of osmotic pumps as a model for biofilms in implant associated infections, we implanted osmotic pumps pre-colonized with bioluminescent *Staphylococcus aureus* Xen29 in C57Bl/6 mice (Figure 1A). We monitored the infection *in vivo* via non-invasive bioluminescent imaging using a Spectrum CT IVIS, a system routinely used to study biofilm development *in vivo*. Its main advantage is its ability to non-invasively and longitudinally record the development of infections *in vivo* recording microbe-encoded luciferase activity. As determined by light radiance (photons/sec/cm^2^/str), all infections showed a characteristic kinetic: Starting with an early expansion of the luminescent bacteria reaching a peak in radiance within the first 48 h post-implantation before it started to progressively decrease until the end of the experiments (Figure 1B). The mice implanted with pre-colonized osmotic pumps displayed some early weight loss (< 10% of bodyweight) but quickly recovered as the infection declined (data not shown).

To validate the existence of bacterial biofilms on the surface of the osmotic pumps, the pumps were harvested after 10 days and first analyzed by immunofluorescence. As shown in Figure 1C, tight clusters of *S. aureus* could be observed on the surface of the osmotic pumps suggesting that the bacteria reside in biofilms. To confirm this observation, we employed scanning electron microscopy (Figure 1D). Indeed, these high resolution images reveal dense clusters of *S. aureus*. In most cases bacteria were also embedded in an extracellular matrix. This became particularly apparent in areas where the bacteria had been stripped during the preparation of the sample. Excavations with the shape of bacterial cells could be found in the matrix (asterisks in Figure 1D). Of note, bacterial attachment to the external surface of the pump is not always stable as some bacterial clusters appeared to be lost during the preparation of the samples. While the other hand some of these cells appear to be so tightly packed against each other that their typical spherical geometry is altered (arrows in Figure 1D).

**Figure 2:**
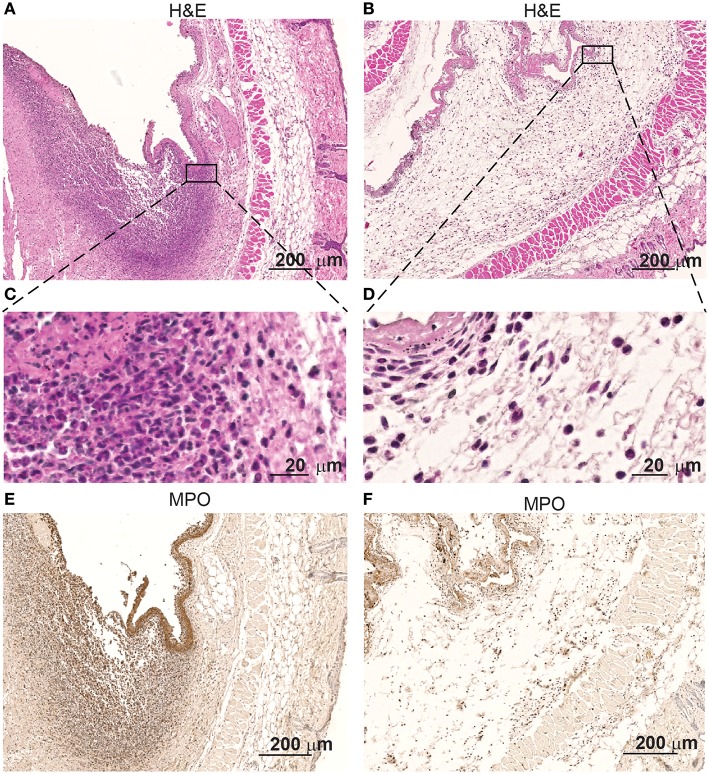
Skin infiltrates increase when in contact with *S aureus* Xen29. Microscopic evaluation at different magnification of skin samples on day 2 after implantation of osmotic pumps colonized **(A,C,E)** or non-colonized with *S aureus* Xen29 **(B,D,F)**. H&E staining **(A–D)** and anti-MPO **(E,F)** immunostaining of tissue surrounding *S. aureus* colonized pumps. These images are representative for 6 mice per experimental condition.

Interestingly, immunohistochemistry of skin samples derived from the surroundings of the colonized pumps at the height of the infection show infiltrates of high numbers of polymorphonuclear cells (Figure 2A). Inflammation of the deep dermis and superficial musculature becomes also apparent at the contact areas with the *S. aureus* colonized pumps. In contrast, no such changes were observed when pumps had not been colonized prior implantation (Figure 2B).

Immunostaining revealed that myeloperoxidase (MPO is expressed by the polymorphonuclear infiltrating cells at the contact interphase of the colonized osmotic pumps and the host tissue (Figure 2C). MPO is a major neutrophil effector protein stored in large amounts in the azurophilic granules and is released into the phagosome after phagocytosis of pathogens. While the majority of MPO remains in the phagosome, up to 30% of total cellular MPO can be released as active enzyme into the extracellular space via degranulation or by the formation of extracellular traps by neutrophil (49). Apparently such neutrophilic granulocytes are attracted to these colonized implants. Only few of such cells were found around non-colonized pumps (Figure 2D).

### Ectopic Application of IL-1β Enhances the Spread of *Staphylococcus aureus* Biofilm *in vivo*

The use of the osmotic pumps allowed us to test the effect of immunologically active soluble compounds on bacterial colonization and biofilm formation. In addition, their effect on the accumulation of immune cells could also be tested. Thus, we pre-colonized osmotic pumps with *S. aureus* Xen29 and filled the pumps prior implantation with various compounds known to affect different cells and arms of the immune system.

**Figure 3:**
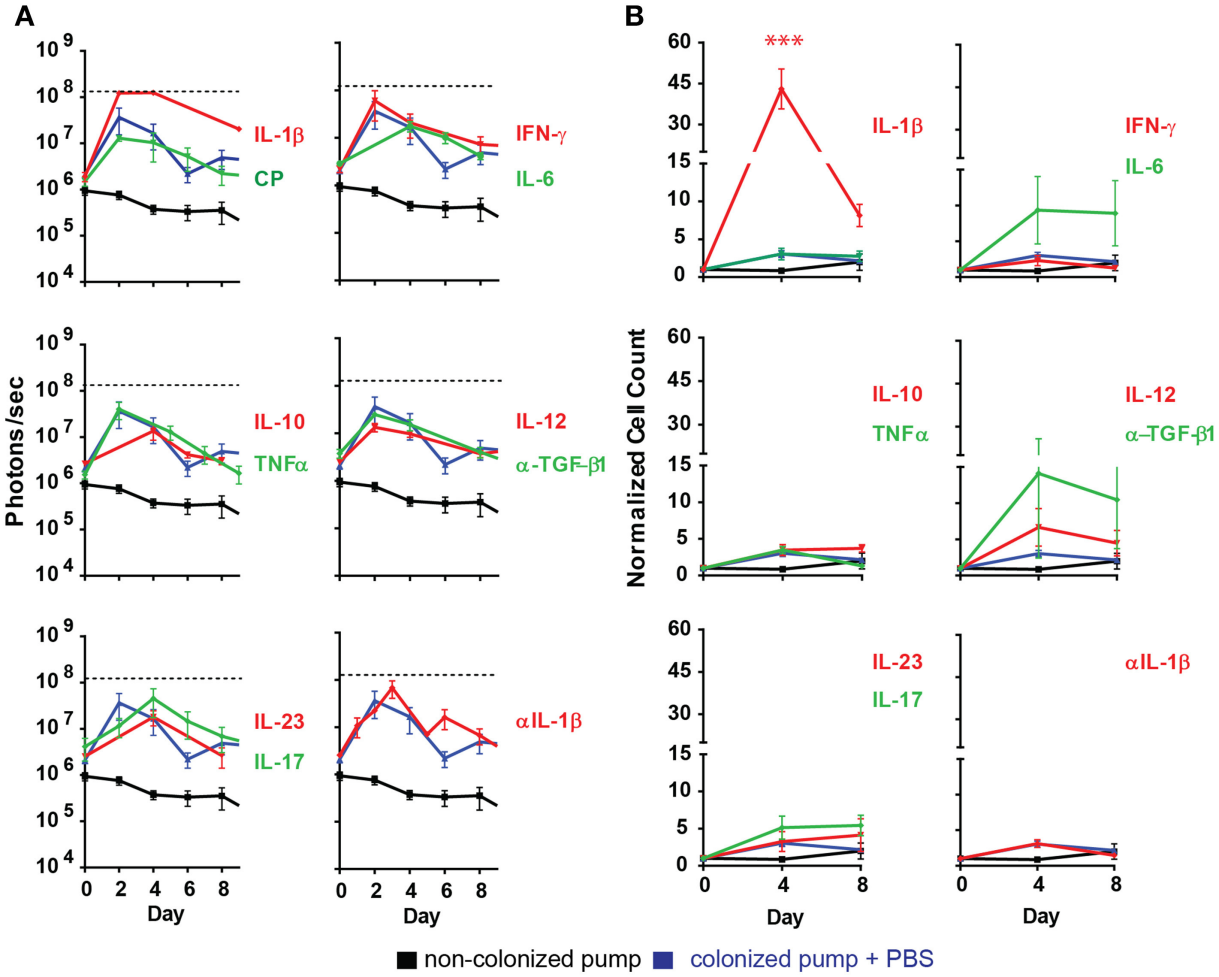
Ectopically supplied Il-1β increases measured luminescent counts. **(A)** Quantification of bioluminescence of *S aureus* Xen29 in C57Bl/6 mice implanted with colonized osmotic pumps (CP). **(B)** Normalized cell counts for cd11b^+^ly6g^+^ cells measured in the blood circulation of C57Bl/6 mice implanted with colonized osmotic pumps. Data is presented as mean ± SD for six mice used in each experimental group during two independent experiments (***p < 0.0002).

Most of the cytokines tested (IL-6, IL-10, IL-12, IL-17, IL-23, IFNγ, TNFα) and anti-TGF-β1 mAb had no significant effect in the development of the bacterial biofilm as revealed by the peak radiance emitted by the *S. aureus* Xen29 (Figure 3A). Likewise, the kinetics of bioluminescence detected were similar to the situation were PBS was applied as control (Figure 3A). In contrast, under the influence of the pro-inflammatory cytokine IL-1β the peak radiance at the height of infection was strongly increased, even exceeding the upper limit of detection over a large area of the skin (Figure 3A). These findings indicate that the pro-inflammatory cytokine IL-1β leads to augmented biofilm formation. We also addressed potential effects of IL-1β on S. aureus Xen 29 by culturing bacteria in the presence of this cytokine. However, we failed to observe any effects on bacterial growth (data not shown). Due to the strong effect of Il-1β *in vivo*, we also loaded the pre-colonized pumps with anti-Il-1β antibodies to block Il-1β activity during biofilm infection. Under these conditions, bacterial colonization was similar to experiments where pumps were filled with PBS indicating that Il-1β is not essential for the maintenance of the biofilm infection. We did not observe a quantitative difference in the local recruitment of neutrophils in the blood or per histological micrographs analyzed.

During the first days of the infection significantly increased numbers of neutrophils (CD11b^+^Ly6G^+^ cells) were also found in the blood of mice implanted with IL-1β filled pumps (Figure 3B) while the effect of other cytokines on neutrophil counts were minimal. It should be noted that blood neutrophil counts were not increased with statistical significance after application of any other cytokines tested (Figure 3B).

**Figure 4:**
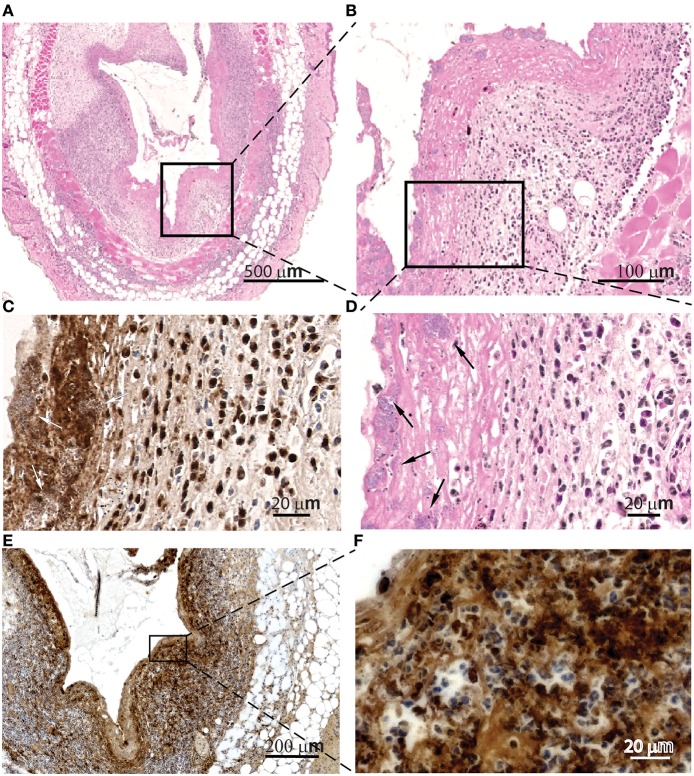
Cellular traps are found in the contact surfaces with *S aureus*. Microscopic evaluation at different magnification of skin samples on day 2 after implantation of osmotic pumps colonized with *Staphylococcus aureus* Xen29 and filled with IL-1β, H&E staining **(A,B,D)** anti-MPO immunostaining **(C)** and anti-histone H3 (citrulline R2 + R8 + R17) immunostaining **(E,F)**. We have also detected *S. aureus* inside cellular traps (arrows). These are representative images for six mice under this experimental conditions.

Histologic evaluation of skin samples neighboring IL-1β-loaded pumps at the peak of infection revealed extracellular MPO lining of the contact regions (Figure 4C) as well as intra- and extracellular citrullinated histones in the tissues surrounding the contact regions with the pumps (Figures 4E,F). We also detected *S. aureus* inside cellular traps (Figure 4, arrows). These observations might indicate the activation of myeloid cells and the formation of NETs. Histologic evaluation of skin samples neighboring PBS-loaded pumps at the peak of infection are depicted in the Supplementary Figure 1.

**Figure 5:**
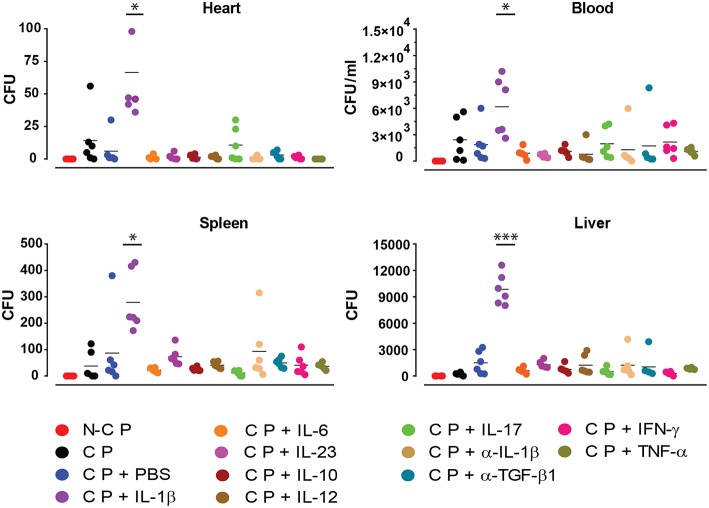
Colony forming unit counts. Absolute bacterial CFU counts in different organs 10 days after implanting non-colonized osmotic pumps (NCP) or colonized osmotic pumps (CP) supplying different cytokines. Data is presented for six mice used in each experimental group during two independent experiments (*p < 0.0332, ***p < 0.0002).

In order to further investigate the effect of the applied cytokines during infection, we determined the presence of *S. aureus* in different organs by quantifying its colony forming units (CFU) (Figure 5). Compared to all other cytokines tested, increased numbers of bacteria were found in blood, liver, spleen and heart under the influence of ectopic IL-1β.

### Neutrophils Confer Early Tissue Protection Against *Staphylococcus aureus* Biofilm Attachment

**Figure 6:**
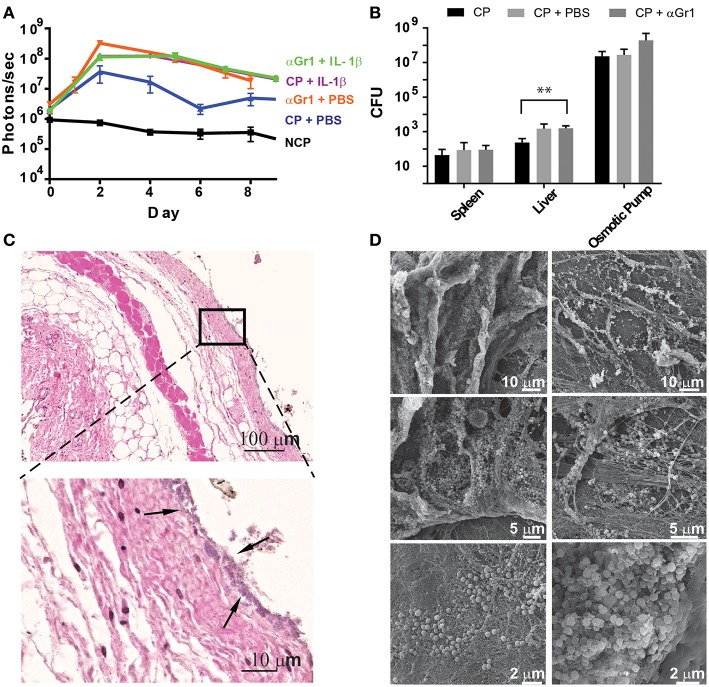
Neutrophils prevent early tissue colonization by *S aureus*. **(A)** Quantification of bioluminescence of *S aureus* Xen29 in C57Bl/6 mice implanted with colonized osmotic pumps, filled with 100 ml of either PBS or IL-1b, with or without neutrophil depletion after anti-Gr-1 mAb application. **(B)** Comparison of absolute bacterial CFU counts in different organs 10 days after implanting colonized osmotic pumps (CP) with or without neutrophil depletion. Data is presented as mean±SD for 6 mice used in each experimental group during 2 independent experiments. **(C)** Microscopic evaluation of skin samples on day 2 after implantation of osmotic pumps colonized with *S aureus* Xen29 into neutrophil-depleted C57Bl/6 mice. Early colonization of skin tissue by *S. aureus* could be detected (arrows). **(D)** Representative examples of scanning electron microscopy from colonized peri-implant tissue. These images are representative for 6 mice per experimental condition. Multiple t-tests were performed with GraphPad PRISM 8 using the Holm-Sidak method, (alpha = 0.05) for comparisons between data for *S. aureus* colonized pumps containing PBS and all other cytokines (* *p* < 0.0332, ** *p* < 0.0021, *** *p* < 0.0002).

Since neutrophils are strongly recruited to the infection site in the presence of IL-1β, we aimed to address their function and effects on bacterial colonization and biofilm formation. Therefore, we depleted C57Bl/6 mice of neutrophils by systemically administering anti-Gr-1 antibodies before implanting osmotic pumps pre-colonized with *S. aureus* Xen29. We confirmed depletion of neutrophils during the first days of the experiment by testing blood for the presence of CD11b^+^Ly6G^+^ cells (data not shown). Under these conditions, increased radiance was detected on osmotic pumps delivering IL-1β or PBS, compared to mice that were not neutrophil depleted (Figure 6A). Bacterial dissemination was also increased in the liver when Gr-1^+^ cells were depleted (Figure 6B).

Histologic analysis at the peak of infection also revealed successful depletion of neutrophils since the strong infiltration of polymorphonuclear cells no longer could be detected at the interface. However, once neutrophils were depleted, early colonization of skin tissue by *S. aureus* could be observed as revealed by a purple-stained band of Gram-positive bacteria spanning the entire contact surface to the pump (Figure 6C). This was not observed in histological images from non-depleted mice. Cellular traps at the contact surface could also no longer be found in neutrophil-depleted mice (Figure 6C). Scanning electron microscopy, 10 days after implantation, confirmed attachment of *S. aureus* to the skin in neutrophil-depleted mice (Figure 6D). These findings indicate that infiltrating neutrophils in non-manipulated mice prevent bacteria from disseminating from the implant's surface into the surrounding tissue. Interestingly, colonization of skin tissue by *S. aureus* appeared to be heterogeneous as the presence of loosely clustered bacteria could sometimes be observed. At the same time some dense clusters, which are compatible with bacteria residing in biofilms, could also be found within the same tissue surrounding the pumps (Figure 6D).

## Discussion

### Establishment of a Mouse Model for *Staphylococcus aureus* Biofilms *in vivo*

We established a simple and reliable mouse model to study the formation of *S. aureus* biofilms on a surface similar to that of a typical medical implant by employing osmotic pumps. In the clinics, biofilms formed by *S. aureus* are found on all kind of medical implants composed of various materials. These include cardiac valves, dental implants, joint replacements as well as catheters. In animal models, catheters are usually employed when studying biofilms. They can be implanted after acquiring particular surface coatings and can be colonized with bacteria post-implantation (32, 33). However, the geometry of catheters differs strongly from those of other devices. In this respect, the colonized lumen of a catheter might represent a rather protected niche and processes controlling biofilm formation in such devices might be substantially different from those on open surfaces. More precisely, the surface of other colonized indwelling devices is constantly exposed to host cells and tissues and in particular to immune effectors of cellular and humoral origin. Besides being representative for indwelling devices, osmotic pumps also allowed us to manipulate ongoing immune reactions during the entire observation period. Via these pumps we ectopically supplied various active cytokines in order to analyze their properties on colonization, biofilm formation as well as their effect on the accumulation and function of immune cells.

In the current setup, the pumps were pre-colonized with bacteria before implantation to yield biofilm formation while bacterial colonization could not be obtained on sterilely implanted pumps when bacteria were administered i.v. post-implantation. We attribute this to the rather short observation period used for these experiments, while in the patient usually month or even years pass before colonization is observed. The use of the recombinant strain *S. aureus* Xen29 proved rather favorable in our studies since its bioluminescent radiance allowed non-invasive monitoring of the colonization of the pumps during the entire observation period.

Under the conditions described in this study, we did not observe variability in the initial attachment of *S. aureus* to the surface of the osmotic pumps and measured an average colonization of 10^7^ CFU over the whole surface by bioluminescent imaging. Formation of biofilms became already evident from the studies using fluorescently-labeled anti-*S. aureus* antibodies revealing dense clusters of bacteria on the surface of the pumps. In the absence of biofilm a dispersed bacterial colonization would be expected. Scanning electron microscopy confirmed the biofilm associated residence of *S. aureus* on the surface of the pumps. Extremely dense clustering of the bacteria was observed and the bacterial extracellular matrix was particularly obvious in samples where the microorganisms had been extracted during the preparation procedure. These images showed deep groves present in the extracellular matrix which apparently had been formed by bacteria. Thus, our implanted pre-colonized osmotic pumps represent a valid model to study biofilm formation by *S. aureus* on the surface of medical devices.

As expected, the infection also attracted myeloid cells. Based on MPO staining as well as the shape of the nuclei a dense ring of neutrophilic granulocytes was formed around the infected pumps. This ring of neutrophils is reminiscent of neutrophil rings around biofilm forming *S. Typhimurium* in solid murine tumors described by us earlier (50). Likewise, the neutrophils in the present study also seemingly fail to phagocytose the S. aureus. It remains currently elusive whether this is due to the biofilm formation of the microorganisms, other escape mechanisms or due to the functional status of the polymorphonuclear cells.

### Ectopic Application of IL-1β Enhances *S. aureus* Biofilm Infection *in vivo*

When using the osmotic pumps to affect host response or biofilm formation, we ectopically applied several pro-inflammatory cytokines as well as IL-10. However, with the exception of IL-1β no effect on biofilm formation could be observed. In the presence of this cytokine, a strong increase in the spread area of *S. aureus* Xen 29 in the tissue surrounding the pumps was also observed. IL-1β is a potent pro-inflammatory cytokine known for mediating acute and chronic local and systemic inflammation (51). When present at high quantities, IL-1β enters the blood stream and stimulates the development of neutrophils and platelets in the bone marrow and promotes their migration and activation. Neutrophil recruitment prompted by IL-1β is known as a physiological requirement for the clearance of *S. aureus* skin infections (52–54). In biofilm infections, *S. aureus* has been shown to express several pore-forming leucocidins (55, 56). In the context of such chronic infections, these toxins are involved in the aggregation of inflammasomes containing NLRP3 and the activation of caspase-1 resulting in expression and release of mature IL-1β (57–59). In contrast to *S aureus* skin infections, implant associated infection where neutrophils and macrophages are already engaged in implant encapsulation represents a different challenge to the organism. In that situation, IL-1β supply led in the present study to increased neutrophil infiltration which, unexpectedly, also resulted in an increase in bacterial load. This finding is reminiscent of bacterial infection by Gram-negative *Pseudomonas aeruginosa* (60, 61) or of *Staphylococcus epidermidis* (32, 33) where IL-1β promotes bacterial survival. It should be noted that the supplied 1 μg/mouse/day IL-1β would result in a theoretical average release of 2 ng IL-1β per gram of body weight per hour. This would be much more than the tens of pg/ml found physiologically in mouse serum and probably accounts for the increased neutrophil recruitment in the blood and in the contact region of our samples.

We speculate that the enhanced infection driven by the ectopic supply of IL-1β might be the result of increased recruitment of neutrophils and macrophages showing a non-phagocytic phenotype similar to leukocytes described by Bhattacharya et al. (62). These authors reported on the inefficacy of such neutrophils to clear the biofilms or ingest biofilm residing *S. aureus in vitro*. Instead, leukocidins secreted by such bacteria were shown to induce strong NETosis indicated by the presence of extracellular elastase and myeloperoxidase. In that study NETosis did not harm the bacteria but rather supported their survival. Although not clarified in detail, it was speculated that the staphylococcal thermonuclease NucA might be responsible for this effect by digesting NET DNA. This nuclease is also expressed by the strain of S. aureus used in this study. Applied to our model, this would suggest that trap formation of host cells by recruitment of neutrophils via IL-1β might indirectly contribute to bacterial spread on the implant's surface. Indeed we believe we have found cell trap formation and NETosis in our model because of the presence of extracellular MPO and citrullinated histones along the tissue lining the contact region of *S. aureus* biofilms. The modification of arginines by peptidyl arginine deiminases in chromatin histones to citrulline is considered a hallmark for NETosis (63).

A role of neutrophils in driving biofilm formation was also confirmed by depleting these cells in mice. Absence of neutrophils not only led to enhanced infection but also to the dissemination of *S. aureus* into the surrounding tissue. Disseminated bacteria also form biofilms in these new niches but the aggregations of bacteria appears more heterogeneous compared to that on pump surfaces. This might be the result of a less homogeneous adhesion of the bacteria to cells or matrix of the surrounding tissues. Importantly, the strong bacterial dissemination into the surrounding tissue in neutrophil-depleted mice indicates that the newly recruited neutrophils surrounding the pumps form a barrier that protects the neighboring tissue, despite being unable to phagocytose the bacteria or clear the biofilms by any other means. However, it does not seem unlikely that factors produced by neutrophils directly or indirectly lead to enhanced biofilm formation. Although the nature of such factors remains currently elusive, they could explain why increased neutrophil recruitment goes along with increased biofilm formation as described in this study.

## Data Availability

Original Fluorescent images, Histological images, electron micrographs and raw data supporting the conclusions of this manuscript can be found under the 10.17632/mb49v25tbv.1 and will be made available by the authors, without undue reservation, to any qualified researcher.

## Ethics Statement

This study was carried out in accordance with the local animal welfare regulations. The protocol was reviewed and approved by the institutional review board and the Niedersächsisches Landesamt für Verbraucherschutz und Lebensmittelsicherheit (LAVES) under the permission number 14–1567.

## Author Contributions

RG, HF, SW, and RF contributed to conception and design of the study. RG performed animal experiments, microbiological methods, and immunohistochemical microscopy. AB performed tissue sample preparation. MR: scanning electron microscopy. RG, SW, and RF wrote the manuscript. All authors contributed to manuscript revision, read, and approved the submitted version.

### Conflict of Interest Statement

After working on this project HF was employed by BioNTech SE. The remaining authors declare that the research was conducted in the absence of any commercial or financial relationships that could be construed as a potential conflict of interest.

## References

[1] European Centre for Disease Prevention and Control Surveillance of Surgical Site Infections in European hospitals – HAISSI Protocol. Stockholm: ECDC (2012). 10.2900/12819

[2] European Centre for Disease Prevention and Control Point Prevalence Survey of Healthcare – Associated Infections and Antimicrobial Use in European Acute Care Hospitals. Stockholm: ECDC (2013). 10.2900/86011

[3] Bernthal NM Stavrakis AI Billi F Cho JS Kremen TJ Simon SI. A mouse model of post-arthroplasty *Staphylococcus aureus* joint infection to evaluate *in vivo* the efficacy of antimicrobial implant coatings. PLoS ONE. (2010) 5:e12580. 10.1371/journal.pone.0012580 20830204 PMC2935351

[4] Chugani SA Whiteley M Lee KM D'Argenio D Manoil C Greenberg EP. QscR, a modulator of quorum-sensing signal synthesis and virulence in Pseudomonas aeruginosa. Proc Natl Acad Sci USA. (2001) 98:2752–7. 10.1073/pnas.051624298 11226312 PMC30211

[5] Kwiecinski J Peetermans M Liesenborghs L Na M Björnsdottir H Zhu X. Staphylokinase control of *Staphylococcus aureus* biofilm formation and detachment through host plasminogen activation. J Infect Dis. (2015) 213:139–48. 10.1093/infdis/jiv360 26136471

[6] Vila J Sáez-López E Johnson JR Römling U Dobrindt U Cantón R. *Escherichia coli*: an old friend with new tidings. FEMS Microbiol Rev. (2016) 40:437–63. 10.1093/femsre/fuw005 28201713

[7] Römling U Galperin MY. Discovery of the second messenger cyclic di-GMP. Methods Mol Biol. (2017) 1657:1–8. 10.1007/978-1-4939-7240-1_1 28889281 PMC5931213

[8] Donlan RM. Biofilms: microbial life on surfaces. Emerging Infect Dis. (2002) 8:881–90. 10.3201/eid0809.020063 12194761 PMC2732559

[9] Waters CM Bassler BL. Quorum sensing : communication in bacteria. Annu Rev Cell Dev Biol. (2005) 21:319–46. 10.1146/annurev.cellbio.21.012704.131001 16212498

[10] Christensen LD Moser C Jensen PØ Rasmussen TB Christophersen L Kjelleberg S. Impact of *Pseudomonas aeruginosa* quorum sensing of biofilm persistence in an *in vivo* intraperitoneal foreign-body infection model. Microbiology. (2007) 153:2312–20. 10.1099/mic.0.2007/006122-0 17600075

[11] Chew SC Kundukad B Seviour T van der Maarel JR Yang L Rice SA. Dynamic remodeling of microbial biofilms by functionally distinct exopolysaccharides. mBio. (2014) 5:e01536-14. 10.1128/mBio.01536-14 25096883 PMC4128364

[12] Gupta S Sharma AK Jaiswal SK Sharma VK. Prediction of biofilm inhibiting peptides: an *in silico* approach. Front Microbiol. (2016) 7:949. 10.3389/fmicb.2016.00949 27379078 PMC4909740

[13] Paharik AE Horswill AR. The staphylococcal biofilm: adhesins, regulation, and host response. Microbiol Spectr. (2016) 4:1–27. 10.1128/microbiolspec.VMBF-0022-2015 27227309 PMC4887152

[14] Römling U Galperin MY. Bacterial cellulose biosynthesis: diversity of operons, subunits, products, and functions. Trends Microbiol. (2015) 23:545–57. 10.1016/j.tim.2015.05.005 26077867 PMC4676712

[15] Bergman P Roan NR Römling U Bevins CL Münch J. Amyloid formation: functional friend or fearful foe? J Intern Med. (2016) 280:139–52. 10.1111/joim.12479 27151743 PMC4956559

[16] Salinas N Colletier JP Moshe A Landau M. Extreme amyloid polymorphism in *Staphylococcus aureus* virulent PSMα peptides. Nat Commun. (2018) 9:3512. 10.1038/s41467-018-05490-0 30158633 PMC6115460

[17] Otto M. Staphylococcal infections: mechanisms of biofilm maturation and detachment as critical determinants of pathogenicity. Annu Rev Med. (2013) 64:175–88. 10.1146/annurev-med-042711-140023 22906361

[18] Dakheel KH Abdul Rahim R Neela VK Al-Obaidi JR Hun TG Yusoff K. Methicillin-resistant *Staphylococcus aureus* biofilms and their influence on bacterial adhesion and cohesion. Biomed Res Int. (2016) 2016:1–14. 10.1155/2016/4708425 28078291 PMC5203895

[19] VanEpps JS Younger JG. Implantable device-related infection. Shock. (2016) 46:597–608. 10.1097/SHK.0000000000000692 27454373 PMC5110396

[20] Müsken M Klimmek K Sauer-Heilborn A Donnert M Sedlacek L Suerbaum S. Towards individualized diagnostics of biofilm-associated infections: a case study. NPJ Biofilms Microbiomes. (2017) 3:22. 10.1038/s41522-017-0030-5 28970943 PMC5620081

[21] Oppong GO Rapsinski GJ Tursi SA Biesecker SG Klein-Szanto AJ Goulian M. Biofilm-associated bacterial amyloids dampen inflammation in the gut: oral treatment with curli fibres reduces the severity of hapten-induced colitis in mice. NPJ Biofilms Microbiomes. (2015) 1:15019. 10.1038/npjbiofilms.2015.19 26855788 PMC4739805

[22] Ingram JP Tursi S Zhang T Guo W Yin C Wynosky-Dolfi AM. A nonpyroptotic IFN-γ-triggered cell death mechanism in nonphagocytic cells promotes salmonella clearance *in vivo*. J Immunol. (2018) 200:3626–34. 10.4049/jimmunol.1701386 29654208 PMC6034694

[23] Vidakovic L Singh PK Hartmann R Nadell CD Drescher K. Dynamic biofilm architecture confers individual and collective mechanisms of viral protection. Nat Microbiol. (2017) 3:26–31. 10.1038/s41564-017-0050-1 29085075 PMC5739289

[24] Simmons M Drescher K Nadell CD Bucci V. Phage mobility is a core determinant of phage-bacteria coexistence in biofilms. ISME J Nat Publ Group. (2018) 12:532–43. 10.1038/ismej.2017.190 29125597 PMC5776469

[25] Müsken M Pawar V Schwebs T Bähre H Felgner S Weiss S. Breaking the vicious cycle of antibiotic killing and regrowth of biofilm-residing *Pseudomonas aeruginosa*. Antimicrob Agents Chemother. (2018) 62:e01635-18. 10.1128/AAC.01635-18 30297365 PMC6256772

[26] Gerstel U Römling U. Oxygen tension and nutrient starvation are major signals that regulate agfD promoter activity and expression of the multicellular morphotype in *Salmonella typhimurium*. Environ Microbiol. (2001) 3:638–48. 10.1046/j.1462-2920.2001.00235.x 11722544

[27] Crull K Rohde M Westphal K Loessner H Wolf K Felipe-López A. Biofilm formation by salmonella enterica serovar typhimurium colonizing solid tumours. Cell Microbiol. (2011) 13:1223–33. 10.1111/j.1462-5822.2011.01612.x. 21507181

[28] Pawar V Crull K Komor U Kasnitz N Frahm M Kocijancic D. Murine solid tumours as a novel model to study bacterial biofilm formation *in vivo*. J Intern Med. (2014) 276:130–9. 10.1111/joim.12258. 24724621

[29] Prabhakara R Harro JM Leid JG Harris M Shirtliff ME. Murine immune response to a chronic *Staphylococcus aureus* biofilm infection. Infect Immun. (2011) 79:1789–96. 10.1128/IAI.01386-10 21282411 PMC3067568

[30] Zhao Y Zhou M Gao Y Liu H Yang W Yue J. Shifted T helper cell polarization in a murine Staphylococcus aureus mastitis model. PLoS ONE. (2015) 10:1–15. 10.1371/journal.pone.0134797 26230498 PMC4521801

[31] Brady RA Mocca CP Plaut RD Takeda K Burns DL. Comparison of the immune response during acute and chronic Staphylococcus aureus infection. PLoS ONE. (2018) 13:1–13. 10.1371/journal.pone.0195342 29596507 PMC5875981

[32] Boelens JJ Dankert J Murk JL Weening JJ van der Poll T Dingemans KP. Biomaterial-associated persistence of *Staphylococcus epidermidis* in pericatheter macrophages. J Infect Dis. (2000) 181:1337–49. 10.1086/315369 10762565

[33] Boelens JJ van der Poll T Zaat SA Murk JL Weening JJ Dankert J. Interleukin-1 receptor type I gene-deficient mice are less susceptible to *Staphylococcus epidermidis* biomaterial-associated infection than are wild-type mice. Infect Immun. (2000) 68:6924–31. 11083815 10.1128/iai.68.12.6924-6931.2000 PMC97800

[34] Thurlow LR Hanke ML Fritz T Angle A Aldrich A Williams SH. *Staphylococcus aureus* biofilms prevent macrophage phagocytosis and attenuate inflammation *in vivo*. J Immunol. (2011) 186:6585–96. 10.4049/jimmunol.1002794 21525381 PMC3110737

[35] Hanke ML Kielian T. Deciphering mechanisms of staphylococcal biofilm evasion of host immunity. Front Cell Infect Microbiol. (2012) 2:62. 10.3389/fcimb.2012.00062 22919653 PMC3417388

[36] Dapunt U Hänsch GM Arciola CR. Innate immune response in implant-associated infections: neutrophils against biofilms. Materials. (2016) 9:1–10. 10.3390/ma9050387 28773509 PMC5503022

[37] Kimura BH Ishibashi T Shikama Y Okano A Akiyama Y Uchida T Interleukin-la (IL-la) induces thrombocytosis in mice: possible implication. J Clin Invest. (1990) 76:2493–500. 2265245

[38] Puri RK Leland P. Systemic administration of recombinant interleukin-6 in mice induces proliferation of lymphoid cells *in vivo*. Lymphokine Cytokine Res. (1992) 11:133–9. Available online at: http://www.ncbi.nlm.nih.gov/pubmed/1391232 1391232

[39] Kaser A Brandacher G Steurer W Kaser S Offner FA Zoller H. Interleukin-6 stimulates thrombopoiesis through thrombopoietin: Role in inflammatory thrombocytosis. Blood. (2001) 98:2720–5. 10.1182/blood.V98.9.2720 11675343

[40] Voorhees JL Tarr AJ Wohleb ES Godbout JP Mo X Sheridan JF. Prolonged restraint stress increases IL-6, reduces IL-10, and causes persistent depressive-like behavior that is reversed by recombinant IL-10. PLoS ONE. (2013) 8:e58488. 10.1371/journal.pone.0058488 23520517 PMC3592793

[41] Gately MK Warrier RR Honasoge S Carvajal DM Faherty DA Connaughton SE. Administration of recombinant IL-12 to normal mice enhances cytolytic lymphocyte activity and induces production of IFN-gamma *in vivo*. Int Immunol. (1994) 6:157–67. Available online at: http://www.ncbi.nlm.nih.gov/pubmed/7908534 7908534 10.1093/intimm/6.1.157

[42] Sonnenberg GF Nair MG Kirn TJ Zaph C Fouser LA Artis D. Pathological versus protective functions of IL-22 in airway inflammation are regulated by IL-17A. J Exp Med. (2010) 207:1293–305. 10.1084/jem.20092054 20498020 PMC2882840

[43] Kleinschek MA Müller U Schütze N Sabat R Straubinger RK Blumenschein WM. Administration of IL-23 engages innate and adaptive immune mechanisms during fungal infection. Int Immunol. (2009) 22:81–90. 10.1093/intimm/dxp117 19951959

[44] Sato K Takahashi N Suda T Prolonged decrease of serum calcium concentration by murine 7-interferon in hypercalcemia human tumor (EC-GI)-bearing nude mice. Cancer Res. (1992) 52:444–9. 1728416

[45] Biesmans S Bouwknecht JA Ver Donck L Langlois X Acton PD De Haes P Peripheral administration of tumor necrosis factor-alpha induces neuroinflammation and sickness but not depressive-like behavior in mice. Biomed Res Int. (2015) 2015:716920 10.1155/2015/716920 26290874 PMC4531164

[46] Dasch JR Pace DR Waegell W Inenaga D Ellingsworth L. Monoclonal antibodies recognizing transforming growth factor-beta. Bioactivity neutralization and transforming growth factor beta 2 affinity purification. J Immunol. (1989) 142:1536–41. Available online at: http://www.ncbi.nlm.nih.gov/pubmed/2537357 2537357

[47] Ueda R Fujita M Zhu X Sasaki K Kastenhuber ER Kohanbash G. Systemic inhibition of transforming growth factor- in glioma-bearing mice improves the therapeutic efficacy of glioma-associated antigen peptide vaccines. Clin Cancer Res. (2009) 15:6551–9. 10.1158/1078-0432.CCR-09-1067 19861464 PMC2783346

[48] Chien CH Lee MJ Liou HC Liou HH Fu WM. Local immunosuppressive microenvironment enhances migration of melanoma cells to lungs in DJ-1 knockout mice. PLoS ONE. (2015) 10:e115827. 10.1371/journal.pone.0115827 25706411 PMC4338246

[49] Parker H Albrett AM Kettle AJ Winterbourn CC. Myeloperoxidase associated with neutrophil extracellular traps is active and mediates bacterial killing in the presence of hydrogen peroxide. J Leukoc Biol. (2012) 91:369–76. 10.1189/jlb.0711387 22131345

[50] Westphal K Leschner S Jablonska J Loessner H Weiss S. Containment of tumor-colonizing bacteria by host neutrophils. Cancer Res. (2008) 68:2952–60. 10.1158/0008-5472.CAN-07-2984 18413765

[51] Dinarello CA. Interleukin-1 beta, interleukin-18, and the interleukin-1 beta converting enzyme. Ann N Y Acad Sci. (1998) 856:1–11. Available online at: http://www.ncbi.nlm.nih.gov/pubmed/9917859 9917859 10.1111/j.1749-6632.1998.tb08307.x

[52] Mölne L Verdrengh M Tarkowski A. Role of neutrophil leukocytes in cutaneous infection caused by Staphylococcus aureus. Infect Immun. (2000) 68:6162–7. 10.1128/IAI.68.11.6162-6167.2000 11035720 PMC97694

[53] Miller LS O'Connell RM Gutierrez MA Pietras EM Shahangian A Gross CE MyD88 mediates neutrophil recruitment initiated by IL-1R but not TLR2 activation in immunity against *Staphylococcus aureus*. Immunity. (2006) 24:79–91. 10.1016/j.immuni.2005.11.011 16413925

[54] Miller LS Inflammasome-mediated production of IL-1 is required for neutrophil recruitment against *Staphylococcus aureus in vivo*. J Immunol. (2007) 179:6933–42. 10.4049/jimmunol.179.10.6933 17982084

[55] Alonzo F Torres VJ. The bicomponent pore-forming leucocidins of *Staphylococcus aureus*. Microbiol Mol Biol Rev. (2014) 78:199–230. 10.1128/MMBR.00055-13 24847020 PMC4054254

[56] Spaan AN van Strijp JAG Torres VJ. Leukocidins: staphylococcal bi-component pore-forming toxins find their receptors. Nat Rev Microbiol. (2017) 15:435–47. 10.1038/nrmicro.2017.27 28420883 PMC5621924

[57] Mariathasan S Weiss DS Newton K McBride J O'Rourke K Roose-Girma M. Cryopyrin activates the inflammasome in response to toxins and ATP. Nature. (2006) 440:228–32. 10.1038/nature04515 16407890

[58] Brodsky IE Monack D. NLR-mediated control of inflammasome assembly in the host response against bacterial pathogens. Semin Immunol. (2009) 21:199–207. 10.1016/j.smim.2009.05.007 19539499

[59] Rapsinski GJ Wynosky-Dolfi MA Oppong GO Tursi SA Wilson RP Brodsky IE. Toll-like receptor 2 and NLRP3 cooperate to recognize a functional bacterial amyloid, curli. Infect Immun. (2015) 83:693–701. 10.1128/IAI.02370-14 25422268 PMC4294241

[60] Palomo J Marchiol T Piotet J Fauconnier L Robinet M Reverchon F. Role of IL-1β in experimental cystic fibrosis upon *P. aeruginosa* infection. PLoS ONE. (2014) 9:e114884. 10.1371/journal.pone.0114884 25500839 PMC4264861

[61] Wonnenberg B Bischoff M Beisswenger C Dinh T Bals R Singh B. The role of IL-1β in Pseudomonas aeruginosa in lung infection. Cell Tissue Res. (2016) 364:225–9. 10.1007/s00441-016-2387-9 26984603

[62] Bhattacharya M Berends ETM Chan R Schwab E Roy S Sen CK. *Staphylococcus aureus* biofilms release leukocidins to elicit extracellular trap formation and evade neutrophil-mediated killing. Proc Natl Acad Sci USA. (2018) 115:7416–21. 10.1073/PNAS.1721949115 29941565 PMC6048508

[63] Wang Y Wysocka J Sayegh J Lee YH Perlin JR Leonelli L. Human PAD4 regulates histone arginine methylation levels via demethylimination. Science. (2004) 306:279–83. 10.1126/science.1101400 15345777

