# Supplementary material for: IL-1β Promotes Staphylococcus aureus Biofilms on Implants in vivo
Source: Front Immunol. 2019 May 17;10:1082. doi: 10.3389/fimmu.2019.01082 (PMC6534041; doi:10.3389/fimmu.2019.01082)
Supplement: Supplementary file 1 [file Data_Sheet_1.docx]

Supplementary Material

# Supplementary Figures and Tables

## Supplementary Figure 1


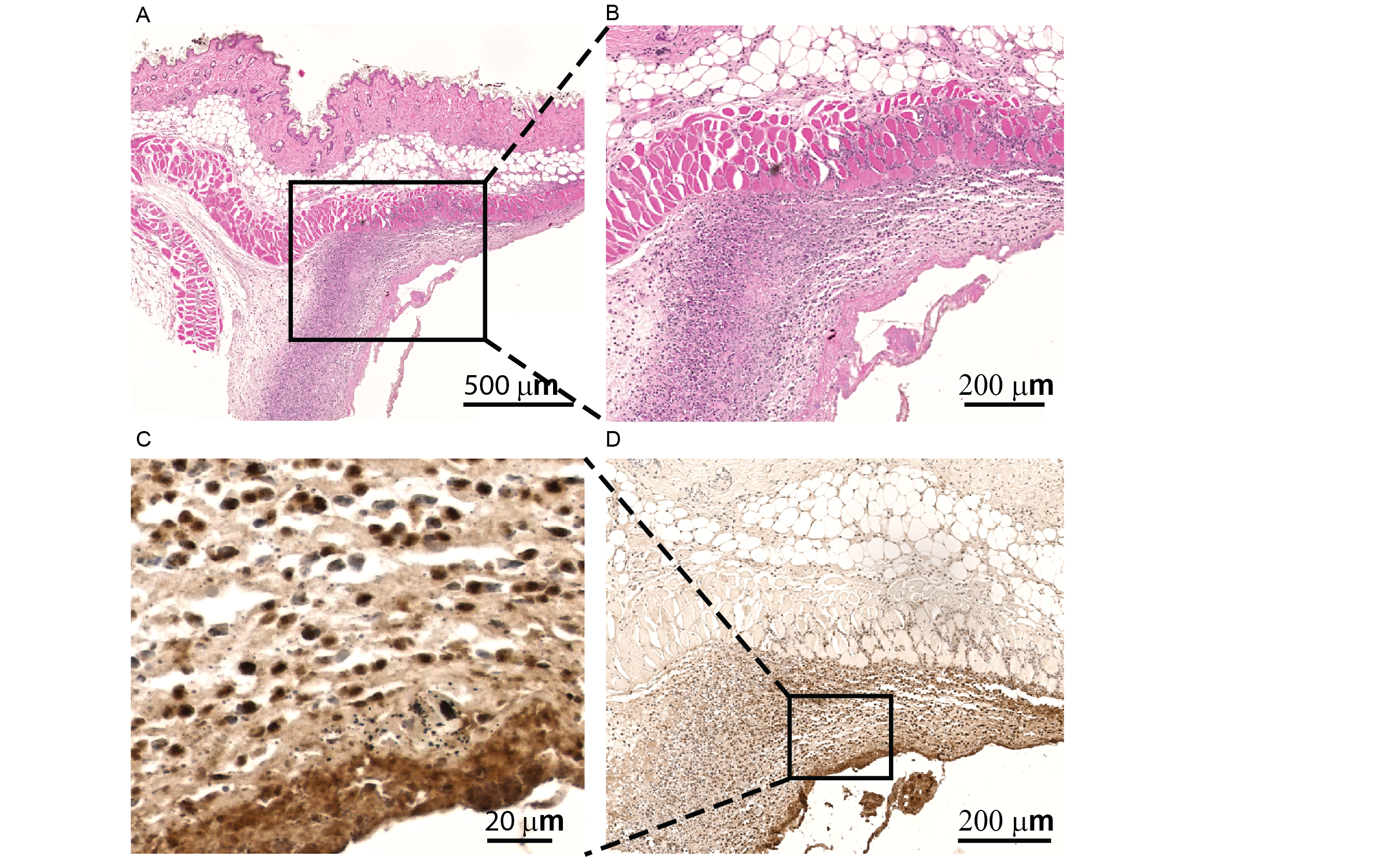


**Supplementary Figure 1.** Microscopic evaluation at different magnification of skin samples on day 2 after implantation of osmotic pumps colonized with *Staphylococcus aureus* Xen29 and filled with PBS, H&E staining (a,b) anti-MPO immunostaining(c,d). These are representative images for 6 mice under this experimental conditions.
